# Disease severity affects knee range of motion but not strength deficits in knee osteoarthritis: a systematic review and meta-analysis

**DOI:** 10.3389/fmed.2026.1737973

**Published:** 2026-02-13

**Authors:** Manca Opara Zupančič, Nejc Šarabon

**Affiliations:** 1 Faculty of Health Sciences, University of Primorska, Izola, Slovenia; 2 Ludwig Boltzmann Institute for Rehabilitation Research, Vienna, Austria

**Keywords:** arthritis, osteoarthritis, knee, rehabilitation, risk factors

## Abstract

**Objectives:**

To compare knee range of motion and muscle strength between individuals with symptomatic knee osteoarthritis and healthy controls, and to assess how Kellgren–Lawrence grade and measurement protocols affect these outcomes.

**Methods:**

A systematic search of PubMed, Scopus, and Web of Science identified studies comparing knee flexion/extension range of motion or flexor/extensor strength between patients with knee osteoarthritis and controls. Risk of bias was assessed with Joanna Briggs Institute tools. Pooled mean and standardized mean differences with 95% confidence intervals were calculated using random-effects meta-analyses.

**Results:**

Thirty studies were included. Compared with healthy controls, individuals with knee osteoarthritis showed significantly reduced knee flexion [MD = 16.30°, 95%CI (11.40, 21.21)] and extension [MD = 4.25°, 95%CI (2.30, 6.19)], with greater flexion loss in advanced KL grades. Knee osteoarthritis participants also demonstrated significantly lower strength across all contraction types: isometric [extensors: SMD = 0.86, 95%CI (0.57, 1.14); flexors: SMD = 0.52, 95%CI (0.30, 0.74)], concentric [extensors: SMD = 1.07, 95%CI (0.65, 1.50); flexors: SMD = 0.77, 95%CI (0.43, 1.12)], and eccentric extensor strength. Strength deficits were consistent across Kellgren–Lawrence grades, knee joint angles, and angular velocities during testing.

**Conclusions:**

Individuals with symptomatic knee osteoarthritis present with marked reductions in knee range of motion and strength. While range of motion impairments worsen with disease severity, strength deficits are stable across Kellgren–Lawrence grades and measurement protocols. Given the very low to low certainty of evidence, results should be interpreted with caution.

## 1 Introduction

Knee osteoarthritis (KOA) is one of the most prevalent forms of osteoarthritis (OA) (1), affecting 16% of individuals aged ≥15 years and 23% of those over 40 years worldwide (2). Clinical guidelines state that a confident diagnosis of KOA can be made based solely on symptoms and signs (3), although radiographic imaging remains a common tool for confirming the presence of KOA and grade severity using the Kellgren–Lawrence (KL) classification (4). KOA represents a considerable physical and psychological burden through pain, stiffness, mobility restrictions, disability, and psychological distress (5–8). Mobility restrictions may be linked to limited knee range of motion (RoM) (9), given that most daily activities require full knee extension and ~110° of flexion (10). In addition, reduced function in people with KOA can result from strength deficits, particularly weakness of the knee extensors and flexors (11–13).

Comparisons between individuals with KOA and healthy controls may help identify functional limitations that contribute to or result from the disease, while also providing important information for diagnostic purposes. To date, no systematic review has comprehensively compared knee RoM between these groups. Existing reviews on muscle impairment are outdated, lack meta-analyses or focus only on hip joint (14, 15), despite newer studies (16–18). Therefore, the aim of our systematic review is to evaluate differences in knee joint strength and RoM between individuals with symptomatic KOA and healthy controls. Furthermore, we aim to compare different population subgroups and determine whether strength and mobility deficits are conditioned by specific factors (e.g., KL grade or by variations in measurement protocols). We hypothesize that individuals with KOA will demonstrate poorer outcomes across all strength and RoM measures compared to healthy individuals. Based on Liu et al. (19), who reported a negative correlation between KOA severity and balance, we hypothesize that RoM and strength deficits will be greater in higher than in lower KL grades. Because tibiofemoral compressive forces peak at ~90° flexion (20), we hypothesize that isometric deficits will be greater at this angle than at others. We also expect smaller isokinetic differences at higher velocities, as KOA patients spend less time in painful positions during faster movements.

## 2 Materials and methods

### 2.1 Search strategy and eligibility criteria

The search was conducted in February 2025 across PubMed, Scopus, and Web of Science using Boolean operators and combinations of terms for knee osteoarthritis (e.g., “knee osteoarthritis,” “knee OA,” “gonarthrosis”) and for healthy status (e.g., “healthy,” “normal,” “asymptomatic,” “without KOA”). Outcome-related keywords (e.g., RoM or muscle strength) were excluded to avoid missing studies that reported these measures only as baseline characteristics. Search strategies for the other databases were adapted accordingly, with no filters applied. No additional restrictions were applied.

The inclusion criteria were defined using the PICOS tool (21):

- P (population): Individuals with symptomatic KOA, with a clearly defined radiographic severity according to the KL grading scale.
- I (Intervention): N/A.
- C (Comparison): Healthy individuals without KOA or any other knee-related conditions.
- O (Outcome): Studies were eligible if they reported quantitative measures of (a) knee joint range of motion, specifically flexion and/or extension, assessed in degrees using objective instruments (e.g., goniometer); and/or (b) muscle strength of the knee extensors and/or flexors, measured under isometric or isokinetic conditions, with contraction type (concentric or eccentric) clearly defined and assessed using validated devices (e.g., isokinetic dynamometer, hand-held dynamometer, or comparable strength testing apparatus).
- S (Study design): Observational studies (cross-sectional, case-control, cohort with healthy controls) and baseline data from clinical trials that included a healthy control group. Only articles published in English were included. Abstracts, conference proceedings, and unpublished studies were not included in this review.

Titles and abstracts were screened for relevance, followed by full-text assessment against predefined PICOS criteria. The literature search was conducted by the first author (M. O. Z.), a trained physiotherapist and PhD researcher experienced in systematic reviews and meta-analyses, under the supervision of the senior author (N.Š.), an experienced researcher in musculoskeletal rehabilitation and systematic review methodology. Reference lists of relevant reviews and included studies were also searched to identify additional articles. Eligible records were imported into Microsoft^®^ Excel^®^ LTSC MSO software for further processing. The included studies were directly relevant to the research question, as they compared individuals with symptomatic knee osteoarthritis to healthy controls and reported quantitative measures of knee range of motion and/or muscle strength. These outcomes were essential for assessing functional impairment and differences in musculoskeletal performance associated with knee osteoarthritis, thereby allowing valid evaluation of the study hypothesis.

### 2.2 Data extraction

Two independent reviewers extracted and coded data. Discrepancies were resolved by consensus to ensure reliability. The extracted data included: (a) Participant characteristics (sex, age, height, weight, BMI); (b) KOA characteristics [symptom presence; compartment involvement (medial or lateral); laterality (unilateral or bilateral); joint location (tibiofemoral or patellofemoral); surgical status (scheduled for knee surgery, e.g., TKA or osteotomy); and KL grade]; (c) Control group characteristics [matching criteria and OA exclusion method (radiographic confirmation or absence of signs and symptoms)]; (d) Measurement procedures for RoM and muscle strength; (e) Outcomes, specifically RoM and muscle strength values for each direction of movement. We sought all results compatible with these outcome domains. In clinical trials that included an intervention, we extracted and analyzed data from measurements taken prior to the intervention (i.e., baseline data). When summary statistics were not reported as mean (M) ± standard deviation (SD), data were converted to this format using established formulae (e.g., from confidence intervals, or median and interquartile range). Data were carefully collected into Microsoft^®^ Excel^®^ LTSC MSO software. If the data were presented in a graphical form, we used WebPlotDigitizer software (version 5.2) to obtain the M and SDs. For missing data, study authors were contacted via e-mail and ResearchGate. A reminder was sent after 14 days, and data were deemed irretrievable if no response followed the second inquiry. Potential confounding factors, such as age, sex, and body mass index, were assessed based on the information provided in the included studies. Most studies reported comparable characteristics between participants with KOA and healthy controls. When differences were present, they were acknowledged in the narrative synthesis.

### 2.3 Assessment of the quality of the included studies and certainty of evidence

The quality of the included studies was identified using the Joanna Briggs Institute (JBI) checklist for analytical cross-sectional studies which assesses study quality based on eight items (22). As the statistical analysis item was not applicable, a maximum of seven items was used: 6–7 “Yes” responses indicated high, 4–5 moderate, and 0–3 low quality. Disagreements were resolved by discussion. Quality assessment was not blinded to study results. To explore potential sources of heterogeneity, subgroup analyses were performed according to Kellgren–Lawrence grade and measurement protocols. Study quality was also considered when interpreting the pooled results.

The certainty of evidence was assessed using the GRADE framework (23), covering risk of bias, imprecision, inconsistency, indirectness, and publication bias. Ratings ranged from high to very low. As most studies were observational, the approach was adapted: certainty was downgraded if >25% of studies fell below quality thresholds, if CIs were wide or total *N* < 300, if heterogeneity was substantial (I^2^ > 50%), if evidence was indirect, or if publication bias was suspected.

### 2.4 Data analysis

The meta-analysis was carried out in Review Manager (Version 5.4, The Cochrane Collaboration, 2020). Means, standard deviations, and sample sizes for both, the KOA and healthy control groups were entered into the meta-analytical model. If data normalized to participant demographics (e.g., weight, BMI, age) were available, these values were included in the analysis. We conducted meta-analyses of continuous outcomes to compare individuals with KOA and healthy controls. A random-effects model with the inverse variance method was used to calculate pooled between-group differences. For outcomes measured using the same units and scales (e.g., RoM in degrees), MDs with 95% CIs were calculated. For outcomes assessed using different measurement instruments or protocols (e.g., muscle strength in N, Nm, Nm/kg, N/BMI, among others) standardized mean differences (SMDs) with 95% CIs were used. Accordingly, the effect sizes representing between-group differences were expressed as MD (for RoM, as it was consistently measured in degrees using a goniometer) and SMD (for muscle strength, as different units and measurement devices were used). Meta-analyses required ≥3 studies, and subgroup analyses ≥2 per subgroup. Where sufficient studies were available, subgroups were formed based on KL grade and on measurement protocols (e.g., knee angle during isometric testing or angular velocity during isokinetic testing). To avoid double-counting participants, data from studies that reported outcomes separately for each KL grade were combined using established formulae for pooling summary statistics across groups (23). In cases where isometric strength was reported at multiple knee angles, we selected the value most commonly used across studies (60 ° for isometric strength of both knee extensors and flexors). Similarly, when concentric strength was reported at multiple angular velocities, we used the value most frequently reported (60 °/s for concentric strength of knee extensors and flexors, or the value closest to 60 °/s when 60 °/s was not available). For meta-analyses comparing concentric strength of knee extensors and flexors across different velocities, we included the higher velocity values within the subgroup representing higher angular speeds. Several studies contributed data to more than one outcome-specific meta-analysis. Consequently, participant numbers overlap across figures representing different outcomes. In subgroup analyses, the same healthy control group was sometimes used as a comparator for multiple disease severity or protocol-specific subgroups. Subgroup comparisons were based on independent effect size estimates, and no participant data were double-counted within any single meta-analysis; therefore, this overlap does not affect the validity of the statistical analyses or the test for subgroup differences. Across analyses involving subgroup stratification, the total number of participants displayed in the forest plots reflects repeated use of the same samples across subgroups and therefore does not represent the number of unique individuals included in the analyses. The exact total number of unique control participants is explicitly reported in the figure captions and is also stated in the Results section.

Statistical heterogeneity was assessed using the I^2^ statistic, interpreted according to Cochrane guidelines (0–30% low, 30–60% moderate, 50–90% substantial, 75–100% considerable). Significance was set at *P* ≤ 0.05. For substantial heterogeneity, sensitivity analyses were performed by excluding studies that differed markedly from the pooled effect.

Study characteristics and risk of bias assessments were tabulated. Results of individual studies and pooled effect sizes were displayed using forest plots generated in Review Manager (RevMan 5.4) where appropriate. Additional syntheses are summarized narratively. Risk of bias due to missing results (reporting bias) was assessed using funnel plots when at least 10 studies were available for a given outcome. Funnel plots were generated in Review Manager (RevMan 5.4) and visually inspected for asymmetry.

## 3 Results

### 3.1 General overview of the search results

**Figure 1:**
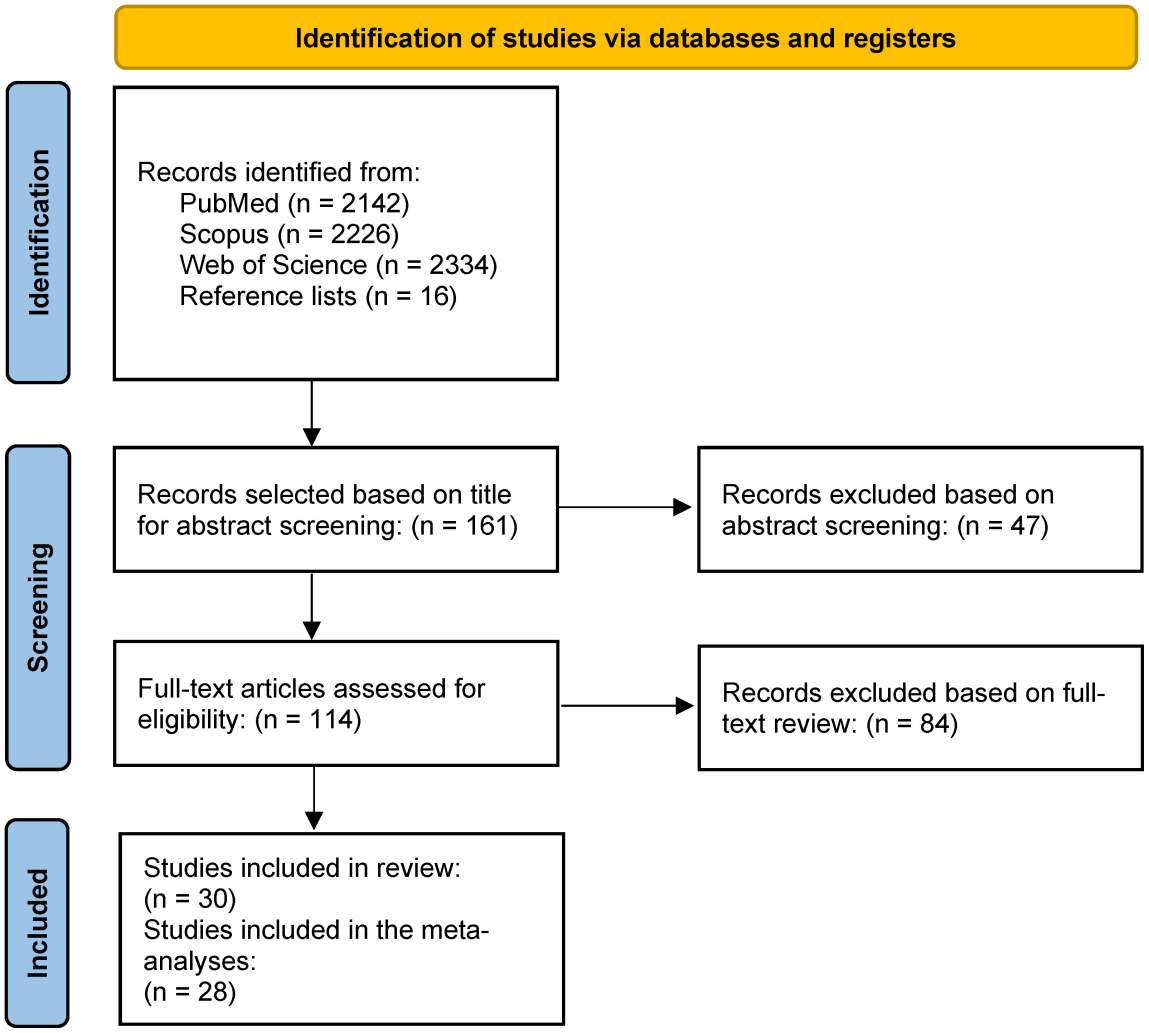
Flowchart of the study search and selection process.

We found 30 studies that were included in the review, 28 of which were eligible for the meta-analysis. Several studies initially appeared to meet the inclusion criteria but were excluded upon full-text review because they did not provide data for healthy control participants, reporting outcomes only for individuals with KOA (24–29). The detailed summary of the search process is shown in Figure 1 (30).

All included studies involved participants with symptomatic KOA, defined either by self-reported pain or by the American College of Rheumatology criteria (31). Some studies examined unilateral KOA (32–34), others bilateral (35, 36) and several included both (11, 17, 37–44). With respect to compartment involvement, some focused on medial KOA (16, 17, 44, 45), others on predominantly medial degeneration (46, 47) while one on both compartments (38). One study included participants awaiting total knee arthroplasty (48). Controls were typically free of KOA, either radiographically or symptomatically. Studies that did not clearly define control status or specify whether the affected leg was tested were included, but sensitivity analyses were performed with and without them. The details of each study are presented in Table 1.

**Table 1:** Characteristics of included studies.

**Author (year)**	**KOA participants**			**Healthy participants**			**Measurement details**	**Results/conclusions**
	**Sample size, sex (M/F)**	**Age (years) in** **mean ±SD**	**KL grade**	**Sample size, sex (M/F)**	**Similar to the KOA subjects in terms of:**	**KOA exclusion method**		
Aily et al. (40)	G1 (middle-aged): *n* = 20 (10M/10F) G2 (older): *n* = 20 (10M/10F)	G1: 45.3 ± 2.7 G2: 74.3 ± 2.8	KL 2–3	G1 (middle-aged): *n* = 20 (10M/10F) G2 (older): *n* = 20 (10M/10F)	Sex, age, BMI	No symptoms, KL 0 or 1	**a) Isometric strength of knee extensors** *Machine:* Biodex system dynamometer (Biodex Medical systems 3 Pro, Shirley, New York, USA) *Starting position:* Seated with knees flexed at 90° *Repetitions and rest:* three maximal contractions held for 3s each, with a 1min rest between contractions **b) Concentric isokinetic strength of knee extensors** *Machine:* Biodex System dynamometer (Biodex Medical Systems 3 Pro, Shirley, New York, USA) *Starting position:* Seated with knees flexed at 90° *Repetitions and rest:* five maximal contractions, 1min rest in between *Speed of movement:* 60°/s	Middle-aged individuals with KOA had significantly lower isometric and concentric torque compared to their healthy participants (*P* < 0.05). Similarly, older adults with KOA also showed significantly lower isometric and concentric torque than healthy older adults (ś < 0.05)
Aily et al. (39)	*n* = 23 (11M/12F)	61.9 ± 9.5	KL 2–3	*n* = 23 (11M/12F)	Sex, age, BMI	No knee pain, KL 0 or 1	**Isometric strength of knee extensors** *Machine:* Isokinetic dynamometer (Multi-Joint System 3, Biodex Medical System, New York, USA) *Starting position:* Seated with knees flexed at 60° *Repetitions and rest:* three maximal contractions held for 3s each, with a 1min rest between contractions	Isometric torque was significantly lower in the KOA group compared to the healthy group (*P* < 0.001) when torque was normalized to body mass. In contrast, the difference in non-normalized isometric torque did not reach statistical significance (*P* = 0.070)
Baert et al. (41)	G1 (early KOA): *n* = 21 (21F) G2 (established KOA): *n* = 24 (24F)	G1 (early KOA): 65.5 ± 7.6 G2 (established KOA): 64.0 ± 7.5	G1 (early KOA): KL 0, 1, or 2^−^ G2 (established KOA): ≥ 2+	*n* = 20 (20F)	Sex, age, weight, height, BMI	Asymptomatic, no history of knee pain, KL 0 or 1	**Isometric strength of knee extensors and flexors** *Machine:* Biodex System 3 Pro (Biodex Medical Systems, New York) *Starting position:* Seated position with 60° and 90° of knee flexion *Repetitions and rest:* Each test was performed three times with maximal contraction held for 5s and 10s rest between trials	Participants with early and established KOA demonstrated significantly lower isometric knee extensor torque at 60° and 90° of knee flexion compared to healthy controls. For knee flexor torque, participants with established KOA showed significantly lower values than controls at both angles, whereas no significant differences were observed between the early KOA group and the control group at either position
Baert et al. (46)	G1 (early KOA): *n* = 14 (14F) G2 (established KOA): *n* = 12 (12F)	G1 (early KOA): 65.4 ± 8.9 G2 (established KOA): 68.3 ± 6.8	G1 (early KOA): KL 0, 1, or 2^−^ G2 (established KOA): ≥ 2+	*n* = 14 (14F)	Sex, age, weight, height, BMI	Asymptomatic, no history of knee pain, KL 0 or 1	**Isometric strength of knee extensors and flexors** *Machine:* Biodex System 3 Pro (Biodex Medical Systems, NY, USA) *Starting position:* Seated position with 60° of knee flexion *Repetitions and rest:* each test was performed three times with maximal contraction held for 5s and 10s rest between trials	The established KOA group showed significantly lower isometric strength in both knee extension (*P* = 0.001) and flexion (*P* = 0.019) compared to the healthy control group. In contrast, the early KOA group had significantly lower isometric extension strength (*P* = 0.007), while their isometric flexion strength was lower than that of healthy controls, but the difference was not statistically significant (*P* = 0.693)
Diraçoglu et al. (35)	*n* = 51 (51F)	55.6 ± 9.7	KL 1–2	*n* = 43 (43F)	Age, BMI, sex	Without clinical or radiological evidence of KOA	**Concentric isokinetic strength of knee extensors and flexors** *Machine:* Biodex System 3-Pro (Biodex Medical Systems, Inc, New York, USA) *Starting position:* Seated, range of motion of the knee was kept at 0°-90° *Repetitions and rest:* Four repetitions for speed of movement of 60°/s and 180°/s and 20 repetitions for speed of movement of 240°/s *Speed of movement:* 60°/s, 180°/s, 240°/s	Patients with KOA exhibited significantly lower isokinetic concentric torque of both the knee flexors and extensors at all tested movement speeds
Gapeyeva et al. (48)	*n* = 10 (10F)	63.0 ± 7.1	KL 3–4	*n* = 10 (10 F)	Sex, age	Without painful joints or any other criteria listed for the patients group	**a) Knee flexion RoM** Active RoM, measured by Gollehon Extendable Goniometer (Lafayette Instrument, USA) **b) Isometric strength of knee extensors** *Machine:* Custom made chair equipped with a chair-fixed standard calibrated strain gauge transducer DST 1778 (Russia) *Starting position:* Seated with knees and hips at 90° and 110°, respectively *Repetitions and rest:* three repetitions of maximal contractions that were held for approximately 3s. A rest period of 2 min was allowed between the attempts	Subjects with KOA had significantly lower RoM compared to healthy controls (*P* < 0.01) and significantly lower isometric strength of the knee extensors (*P* < 0.001)
Unver Kocak et al. (49)	G1: *n* = 33 (8M/25F) G2: *n* = 30 (4M/26F) G3: *n* = 59 (11M/48F) G4: *n* = 61 (2M/59F)	G1: 50.9 ± 11.0 G2: 59.6 ± 9.0 G3: 65.7 ± 9.2 G4: 66.9 ± 7.7	G1: KL1 G2: KL2 G3: KL 3 G4: KL 4	*n* = 35 (20M/15F)	/	No history of knee pain or other symptoms	**Knee flexion RoM** Active RoM measured by universal goniometer	There were no significant differences in knee flexion RoM between healthy subjects and individuals with KOA of KL grades 1 and 2 (*P* > 0.05). However, healthy subjects had significantly higher values compared to those with KL grades 3 and 4 (*P* < 0.000)
Kumar et al. (45)	*n* = 16 (8M/8F)	65.2 ± 9.5	KL ≥ 2	*n* = 12 (6M/6F)	Age, weight, BMI	KL ≤ 1	**Isometric strength of knee extensors** *Machine:* Isokinetic dynamometer (Kin Com Isokinetic International. Harrison, TN 37341) *Starting position:* Knee flexed to 90°*Repetitions and rest:* Three maximal voluntary isometric contractions	The KOA group had lower quadriceps strength but it was not statistically significant (*P* = 0.16)
Liikavainio et al. (11); Lyytinen et al. (79)	G1: *n* = 12 (12M) G2: *n* = 15 (15M) G3: *n* = 19 (19M) G4: *n* = 8 (8M)	G1: 57.7 ± 5.8 G2: 58.7 ± 5.8 G3: 59.1 ± 5.1 G4: 61.2 ± 4.1	G1: KL 1 G2: KL2 G3: KL3 G4: KL4	*n* = 53 (53M)	Age, sex	No Knee OA according to the clinical criteria of the American College of Rheumatology (31)	**a) Knee flexion and extension RoM** Active knee flexion and passive knee extension RoM measured in a supine position with a standard goniometer **b) Isometric strength of knee extensors and flexors** *Machine:* Calibrated dynamometer *Starting position:* sitting position with knee and hip angles fixed at 70° *Repetitions and rest:* as many maximal actions until the peak value no longer increased	The control subjects showed significantly higher knee extension torque values (*P* < 0.01), knee flexion torque (*P* = 0.024), as well as greater knee flexion (*P* < 0.01) and extension (*P* < 0.01) RoM compared to all subjects with KOA
Ling et al. (51)	*n* = 21 (10M/11F)	65.49 ± 2.8	KL 1–4	*n* = 18 (12M/6F)	Age, sex	No knee symptoms, KL0	**Isometric strength of knee extensors** *Machine:* KIN-Com 125E dynamometer (Chattecx, Chattanooga, TN) *Starting position:* knee angle of 60° of flexion *Repetitions and rest:* three trials	Participants with KOA exhibited lower isometric knee extensor strength than controls (*P* = 0.016)
Rodriguez-Lopez et al. (18)	*n* = 18 (18F)	69.6 ± 7.3	KL 2–4	*n* = 26 (26F)	Age, sex, BMI	Asymptomatic and no history of knee pain	**Isometric strength of knee extensors** *Machine:* biodex Medical System 3 dynamometer (Biodex Medical Systems, Shirley, NY) *Starting position:* Knee joint angle of 90° *Repetitions and rest:* four 5s maximal voluntary isometric ramp contractions, separated by 20s rest interval	KOA subjects showed lower isometric strength compared to control subjects (*P* = 0.001)
Sanchez-Ramirez et al. (52)	G1 (early KOA): *n* = 14 (14F) G2 (established KOA): *n* = 19 (19F)	G1: 70.4 ± 4.6 G2: 68.37 ± 6.7	G1: KL 1 G2: KL ≥ 2	*n* = 14 (14F)	Age, sex, BMI, weight, height	No history of knee symptoms or characteristics associated with knee OA, KL 0	**Isometric strength of knee extensors and flexors** *Machine:* biodex System 3 Pro (Biodex Medical System, Shirley, NY, USA) *Starting position:* measured in 60° of flexion position *Repetitions and rest:* three trials of each leg	Control subjects demonstrated significantly higher isometric knee flexor torque compared to the established KOA group (*P* = 0.011). However, no significant differences were observed between the control group and the early KOA group for knee flexor strength (*P* = 0.217). There were also no significant differences in isometric knee extensor torque among the groups (*P* = 0.362)
Rutherford et al. (47)	G1: *n* = 38 (27M/11F) G2: *n* = 33 (28M/5F) G3: *n* = 11 (6M/5F)	G1: 56 ± 8 G2: 59 ± 8 G3: 59 ± 8	G1: KL2 G2: KL3 G3: KL4	*n* = 35 (16M/19F)	Age	Asymptomatic	**Isometric strength of knee extensors and flexors** *Machine:* *Starting position:* knee extension strength was measured at 45° of knee flexion in a seated position, and knee flexion strength at 55° of knee flexion in the same position *Repetitions and rest:* two three second maximal isometric contractions were completed for each muscle group. A minimum 60s rest period separated each contraction	Asymptomatic individuals had greater quadriceps strength than the KL 2 group (*P* < 0.05). Other differences between groups were not significant
Tan et al. (53)	*n* = 30 (30F)	63 ± 6.85	KL 2–3	*n* = 30 (30F)	Sex	/	**Isometric strength of knee extensors and flexors** *Machine:* cybex-350 isokinetic dynamometer system (Lumex Inc., Ronkokoma, New York) *Starting position:* at 30° and 60° of knee flexion *Repetitions and rest:* six maximal tries were made at each flexion degree, with resting interval of 20s between each try	Isometric maximum peak torque loss of knee flexors and extensors was found in KOA group with respect to controls
Vårbakken et al. (37)	*n* = 31 (15M/16F)	55.3 ± 8.0	KL 2–4	*n* = 28 (10M/18F)	Sex, BMI, height, weight	Without knee pain or knee complaints	**Concentric isokinetic strength of knee extensors and flexors** *Machine:* biodex System 4 Dynamometer (Biodex Medical Systems, NY, USA) *Starting position:* seated, with back rest tilted 70° *Repetitions and rest:* five consecutive maximum strength tests *Speed of movement:* 60°/s	There was a significant difference in knee extensor torque between groups (*P* = 0.012), while no significant differences were observed in knee flexor torque (*P* = 0.114)
Yagi et al. (17)	*n* = 22 (22F)	69.5 ± 5.4	KL 2–4	*n* = 15 (15F)	Age, sex, height	Asymptomatic	**a) Knee flexion and extension RoM** Passive knee RoM was measured in flexion and extension with a goniometer **b) Isometric strength of knee extensors** *Machine:* dynamometer Biodex System 4, Biodex Medical Systems, Inc *Starting position:* seated, with 45° of knee flexion *Repetitions and rest:* two repetitions of maximum voluntary contraction for 5s	Healthy controls exhibited significantly greater knee RoM in both flexion (*P* = 0.003) and extension (*P* = 0.024). Isometric MVC torque was also significantly greater in the control group (*P* < 0.001)
Yang et al. (32)	*n* = 18 (14M/4F)	63.88 ± 6.5	KL 2–3	*n* = 18 (13M/5F)	Age, sex, BMI, weight, height	/	**Concentric isokinetic strength of knee extensors and flexors** *Machine:* biodex isokinetic dynamometer (System 4, Biodex Inc., Shirley, NY, United States) *Starting position:* seated *repetitions and rest:* the concentric/concentric pattern was repeated 3 times, with 5 min break between each set of tests *Speed of movement:* 60°/s, 120°/s, 180°/s	Compared to healthy controls, KOA patients had significantly smaller peak torque at all speeds (*P* < 0.05)
Baker et al. (16)	*n* = 20 (10M/10F)	62 ± 7	KL 1–3	*n* = 20 (11M/9F)	Age, height	Asymptomatic	**Isometric strength of knee extensors and flexors** *Machine:* a Human Norm Isokinetic Dynamometer (Computer Sports Medicine Inc., USA) *Starting position:* at 45° of knee flexion *Repetitions and rest:* two, 3s MVIC trials were completed, separated by 40s of rest	No significant between group strength differences were found for knee extensors (*P* = 0.195) and flexors (*P* = 0.067)
Childs et al. (43)	*n* = 24 (56%F)	62 ± 10	KL ≥ 2	*n* = 24 (56%F)	Age, sex, height	No history of knee OA, KL ≤ 1	**Knee flexion and extension RoM** /	Subjects with KOA had significantly less flexion and extension knee RoM (*P* < 0.01) compared to healthy individuals
Emrani et al. (50)	*n* = 20 (M and F)	44.6 ± 2.3	KL 1–2	*n* = 20 (M and F)	Age, weight, height, activity level	No clinical or radiological sign of KOA	**a) Concentric isokinetic strength of knee extensors and flexors** *Machine:* biodex System 2 isokinetic dynamometer (Biodex Medical System, Shirley, NY, USA) *Starting position:* Seated with a backrest at 90° angle. During the test subjects pushed the lever arm of the device through the RoM between 10° and 90° of knee flexion *Repetitions and rest:* two sets of tests, in order of speed. Each test consisted of a continuous maximal flexion-extension and was repeated five times. A 1 min rest was allowed between each two sets of tests, and a 3 min rest was given after each angular speed. A 20 min rest was allowed between the two legs *Speed of movement:* 90°/s, 150°/s **b) Knee flexion and extension RoM** Measured with goniometer while the subject was supine with the hip extended	There were significant differences between the two groups with regard to isokinetic torque at both angular speeds for knee flexors and extensors (*P* < 0.00). There were no significant differences in the values of lower extremity RoM (*P* > 0.05)
Hortobágyi et al. (36)	*n* = 20 (5M/15F)	57.5 ± 7.3	KL ≥ 2	*n* = 20 (5M/15F)	Sex	No knee OA, no knee pain	**a) Isometric strength of knee extensors** *Machine:* Dynamometer (Kin-Com, AP125; Chattecx Inc, Chattanooga, TN) *Starting position:* 1.14 radians of knee flexion *Repetitions and rest:* subjects performed three maximal isometric effort 5s trials with 1 min of rest between trials **b) Concentric and eccentric isokinetic knee extensors strength** *Machine:* Dynamometer (Kin-Com, AP125; Chattecx Inc, Chattanooga, TN) Starting position: *Repetitions and rest:* subjects performed three maximal effort eccentric and concentric isokinetic quadriceps contractions. Each quadriceps contraction was followed by a hamstring contraction with a 1s pause between the efforts. There was 1min of rest between conditions *Speed of movement:* 1.57 radians per second	Overall, KOA patients produced 63% less quadriceps force than control subjects (*P* < 0.05)
Lohnes et al. (34)	*n* = 16 (6M/10F)	61 ± 6	KL 0–3	*n* = 16 (6M/10F)	Age, sex	Asymptomatic	**Isometric strength of knee flexors and extensors** *Machine:* Human Norm Isokinetic Dynamometer (Computer Sports Medicine Inc., USA) *Starting position:* 45° of knee flexion *Repetitions and rest:* two, three second maximal isometric contractions with a 40s rest period separated each contraction	There were no significant differences between KOA and healthy control group for knee flexion (*P* = 0.187) and knee extension (*P* = 0.217) strength
Noehren et al. (54)	*n* = 24 (14M/10F)	60.2 ± 5.5	KL 2–3	*n* = 15 (5M/10F)	Activity, age, BMI	KL ≤ 1 in both knees and no evidence of patellofemoral OA	**Concentric isokinetic knee extensor strength** *Machine:* HUMAN NORM isokinetic dynamometer (CSMi, Stoughton, MA, USA) *Starting position:* Seated, knee extensor strength was tested through a joint arc from 90° to 30° of knee flexion *Repetitions and rest:* five trials spaced by rest period of 30–60s *Speed of movement*	KOA subjects had significantly weaker quadriceps than the control subjects (*P* = 0.003)
Rice et al. (75)	*n* = 15 (7M/8F)	63.0 ± 9.7	KL 2–4	*n* = 15 (7M/8F)	Age, sex, height, mass	No history of knee injury or pathology	**Isometric strength of knee extensors and flexors** *Machine:* *Starting position:* *Repetitions and rest:* three quadriceps MVCs (6s) followed by three hamstring MVCs (6s) with a 2 min rest period between each maximum-effort contraction	Quadriceps peak torque was significantly lower in the KOA group compared with the control group (*P* = 0.005). While hamstrings peak torque was lower in the KOA group compared with the control group, this difference did not reach statistical significance (*P* = 0.101)
Serrao et al. (55)	*n* = 22 (22M)	52 ± 8.1	KL 1–2	*n* = 18 (18M)	Sex	No joint disorders, no radiographic alterations, KL = 0, no history of lower limb pain, illness, injury, trauma or fracture	**Concentric and eccentric isokinetic knee extensor strength** *Machine:* Isokinetic dynamometer (Biodex Multi-Joint System 3, Biodex Medical Inc, Shirley, NY) *Starting position:* Seated with knee flexed at 90°. Range of motion: from 20° to 90° *Repetitions and rest:* five maximal voluntary concentric and five maximal voluntary eccentric contractions at an angular speed of 90°/s and 10 maximal concentric and 10 maximal eccentric contractions at an angular speed of 180°/s. The rest between each type of contraction and velocities lasted for 5 min. *Speed of movement:* 90°/s and 180°/s	In the analysis of concentric knee extensor strength, no differences were found between the groups at any angular speed (*P* = 0.73 at 90°/s; *P* = 0.97 at 180°/s). For eccentric knee extensor strength, differences were found between the groups at 90°/s (*P* = 0.01) and at 180°/s (*P* = 0.04), with higher values for the control group
Teoli et al. (42)	*n* = 22 (6M/16F)	60 ± 7	KL 1–4	*n* = 22 (6M/16F)	Age, sex, BMI	No current lower extremity pain, no diagnosis of lower extremity OA	**Isometric knee extensor strength** *Machine:* Isokinetic dynamometer (Humac Cybex NORM, Computer Sports Medicine Inc., Massachusetts, US) *Starting position:* Seated with 45° of knee flexion *Repetitions and rest:* two 5s MVIC trials with a 30s rest period between trials	No significant differences were observed in knee extensor strength between groups (*P* = 0.335)
Ucurum et al. (33)	*n* = 35 (35F)	59.63 ± 7.81	KL 2–3	*n* = 35 (35F)	Age, sex, weight, height, BMI	No current pain and no OA symptoms	**Isometric knee flexor and extensor strength** *Machine:* Digital hand-held dynamometer (Lafayette Model-01165, Lafayette Instrument Company, Lafayette IN, USA) *Starting position:* Seated with a tight-body angle of 90° and knee flexion of 60° *Repetitions and rest:* three repetitions with a 30s period of rest in between	Extensor strength in KOA group was lower than extensor strength of dominant and nondominant knees of the controls and the effect sizes were moderate (*P* = 0.037 and *d*=0.51 for dominant knee; *P* = 0.039 and *d*=0.50 for non dominant knee). No significant difference was found between contralateral and ipsilateral knees and groups for knee flexor strength (*P* > 0.05)
Yagi et al. (17)	*n* = 18 (18F)	69.7 ± 5.9	KL 2–4	*n* = 22 (22F)	Age, height, weight, BMI	No knee pain	**Knee flexion and extension RoM** Measured in supine using a two-arm goniometer (Sakai Medical Co., Ltd.)	Healthy control group had significantly more knee extension and flexion RoM compared to control group (*P* < 0.001)
Zhang et al. (3)	*n* = 25 (25F)	64.42 ± 2.95	KL 1–3	*n* = 22 (22F)	/	/	**Concentric and eccentric isokinetic knee extensor strength** *Machine:* Multi-joint dynamometer (Con-trex multi joint model, CMV AG, Dubendorf, Switzerland) *Starting position:* Seated *Repetitions and rest:* three repetitions *Speed of movement:* 90°/s	Compared with the control group, the KOA exhibited lower absolute peak knee extension torque, relative peak knee extension and relative flexion torque

n, numerus; KOA, knee osteoarthritis; OA, osteoarthritis; G, group; M, mean or male; SD, standard deviation; F, female; KL, Kellgren-Lawrence Classification System; RoM, range of motion; MVC, maximal voluntary contraction; MVIC, Maximal voluntary isometric contraction; min, minute; s, second.

### 3.2 Assessment of the quality of the included studies

As shown in Supplementary Table S1, the methodological quality of the included studies was predominantly high, with 27 of 30 studies meeting 6–7 JBI criteria and the remaining two classified as moderate; no study was rated low quality.

### 3.3 Differences in knee flexion range of motion between individuals with knee osteoarthritis and healthy controls

**Figure 2:**
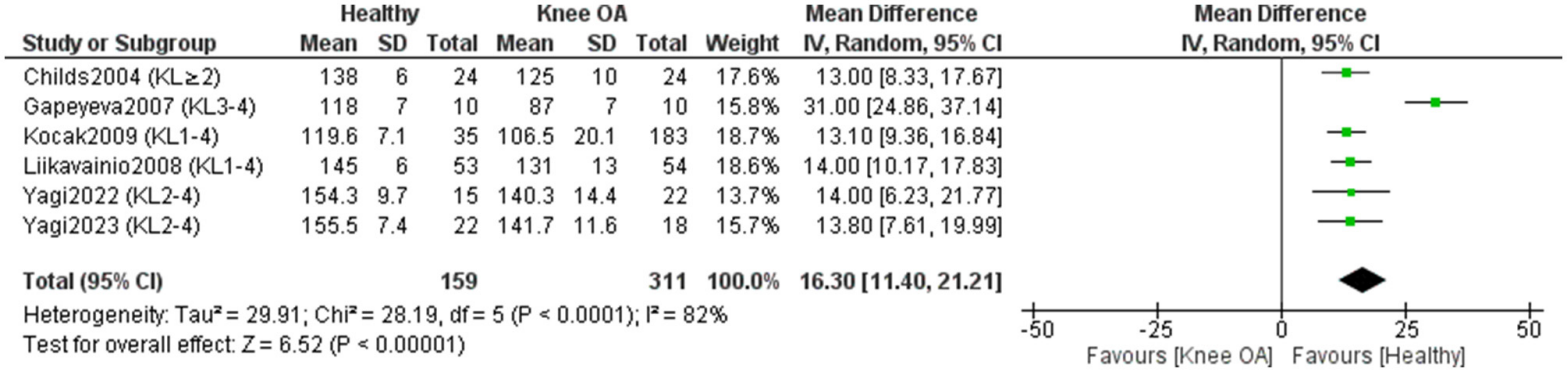
Meta-analysis of differences in knee flexion range of motion between individuals with kne osteoarthritis and healthy controls. Mean differences (MD) are expressed in degrees (°).

Six studies (311 KOA; 159 controls) (11, 17, 43, 44, 48, 49) investigated knee flexion RoM differences (Figure 2). Healthy individuals showed 16.30° greater flexion than those with KOA [MD = 16.30, 95% CI (11.40–21.21), *P* < 0.0001], though heterogeneity was high (I^2^ = 82%). Excluding studies with unclear measurement or control status (43, 49) increased the difference to 18.19 ° and heterogeneity to I^2^ = 87% (*P* < 0.0001). Certainty of evidence was very low, despite all studies being rated high quality.

One study (50) also assessed flexion but lacked values for inclusion; it reported no significant differences (*P* > 0.05) in lower extremity RoM between patients with KOA and healthy controls.

**Figure 3:**
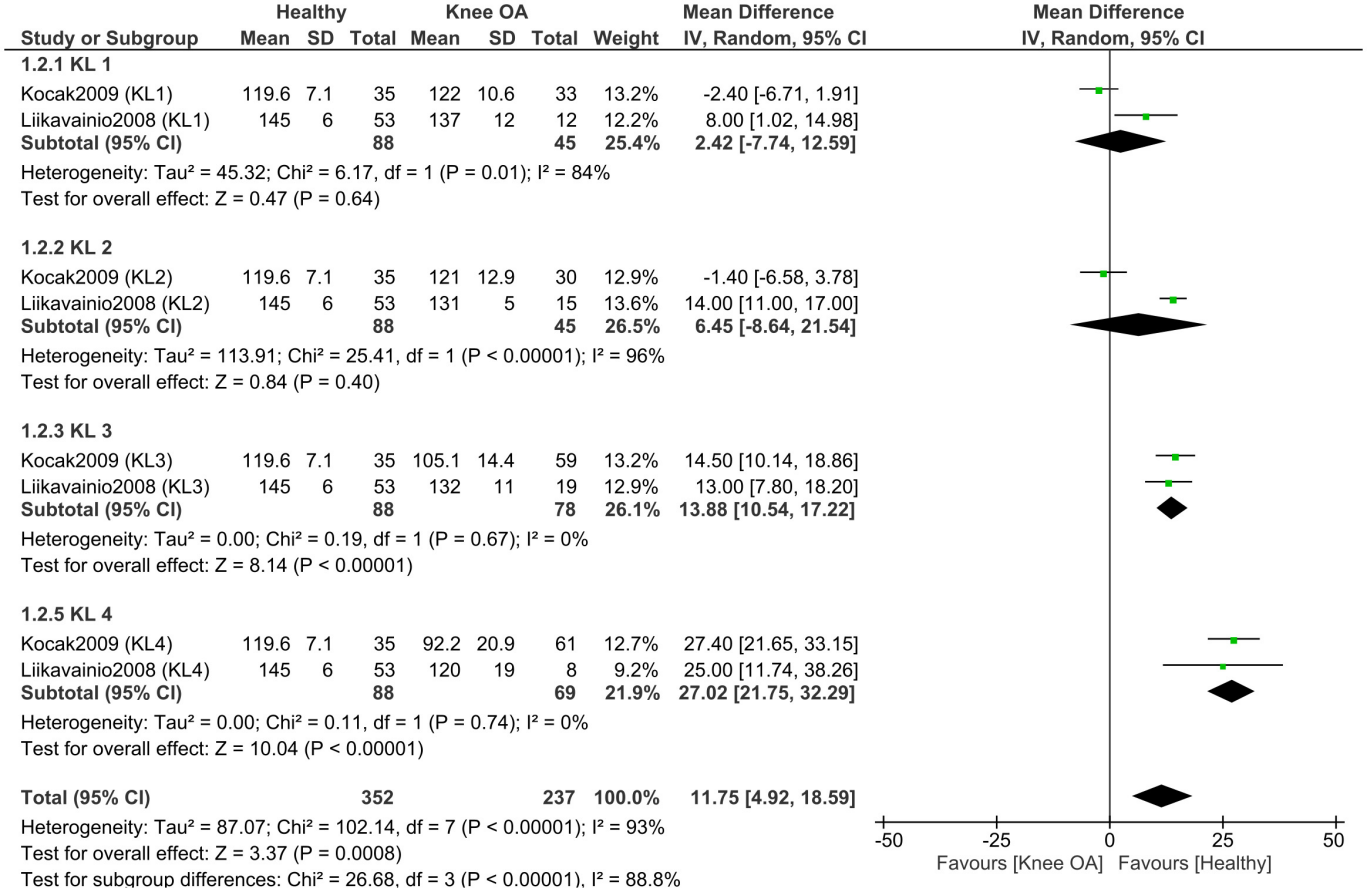
Meta-analysis of knee flexion range of motion differences according to Kellgren–Lawrence grades of osteoarthritis severity. Mean differences (MD) are expressed in degrees (°). The total number of controls displayed in the forest plot reflects repeated use of the same control samples across Kellgren–Lawrence grade subgroups; the number of unique healthy control participants included in this analysis was 88.

To explore the impact of osteoarthritis severity, we conducted a separate meta-analysis (two studies, 237 KOA; 88 controls) (11, 49) comparing knee flexion RoM between healthy controls and individuals with KOA, stratified by KL grade (Figure 3). The results showed significant subgroup differences (*P* < 0.00001), indicating that the magnitude of the difference in knee flexion RoM between healthy individuals and those with KOA depends on the KL grade. When examining the overall effect within each subgroup, significant differences between healthy individuals and those with KOA were found only in KL grade 3 (*P* < 0.00001) and KL grade 4 (*P* < 0.00001). In contrast, no significant differences were observed between healthy individuals and those with KL grade 1 (*P* = 0.64) or KL grade 2 (*P* = 0.40). Moreover, one study (49) reported greater knee flexion in individuals with KL grades 1 and 2 compared to healthy controls. The largest difference in knee flexion RoM was observed between healthy individuals and those with KOA classified as KL grade 4, with a mean difference of 27.02° in favor of the healthy group [MD = 27.02, 95% CI (21.75, 32.29), *P* < 0.00001].

### 3.4 Differences in knee extension range of motion between individuals with knee osteoarthritis and healthy controls

**Figure 4:**
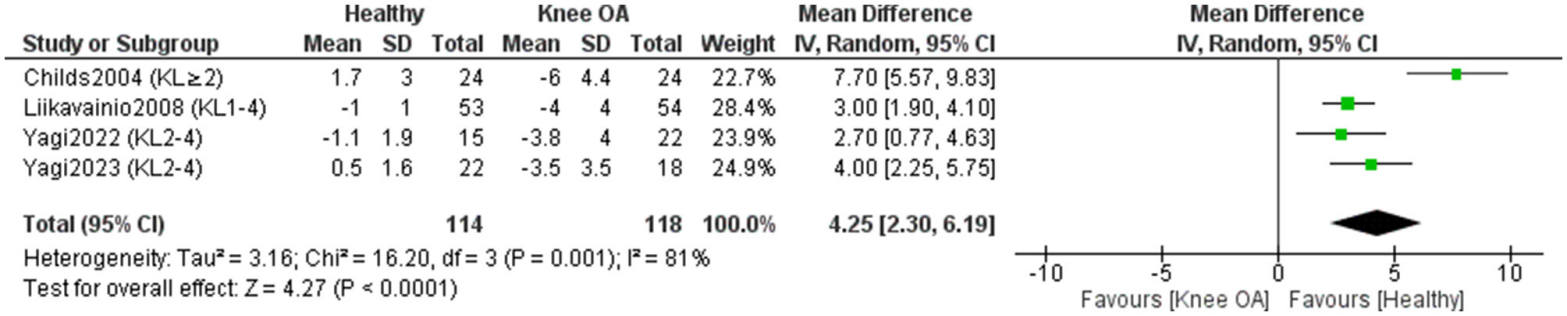
Meta-analysis of differences in knee extension range of motion between individuals with knee osteoarthritis and healthy controls. Mean differences (MD) are expressed in degrees (°).

Four studies included in the meta-analysis (118 KOA; 114 controls) (11, 17, 43, 44) investigated differences in knee extension RoM between individuals with KOA and healthy subjects (Figure 4). Overall, healthy individuals demonstrated 4.25° greater knee extension RoM compared to those with KOA [MD = 4.25, 95% CI (2.30, 6.19), *P* < 0.0001]. However, study effects were highly heterogeneous (I^2^ = 81%). After repeating the meta-analysis with the exclusion of studies that did not clearly report whether measurements were performed on the affected leg in KOA participants or did not explicitly state that control participants were free of KOA (43), heterogeneity decreased to I^2^ = 0%. The between-group difference decreased to 3.17°, remaining statistically significant [MD = 3.17, 95% CI (2.33, 4.01), *P* < 0.00001]. Certainty of evidence was very low, despite all studies being rated high quality.

One study (50) assessed knee extension RoM but lacked measurement values and was excluded from the meta-analysis; it reported no significant differences (*P* > 0.05) between KOA patients and controls.

### 3.5 Differences in isometric knee extensor strength between individuals with knee osteoarthritis and healthy controls

**Figure 5:**
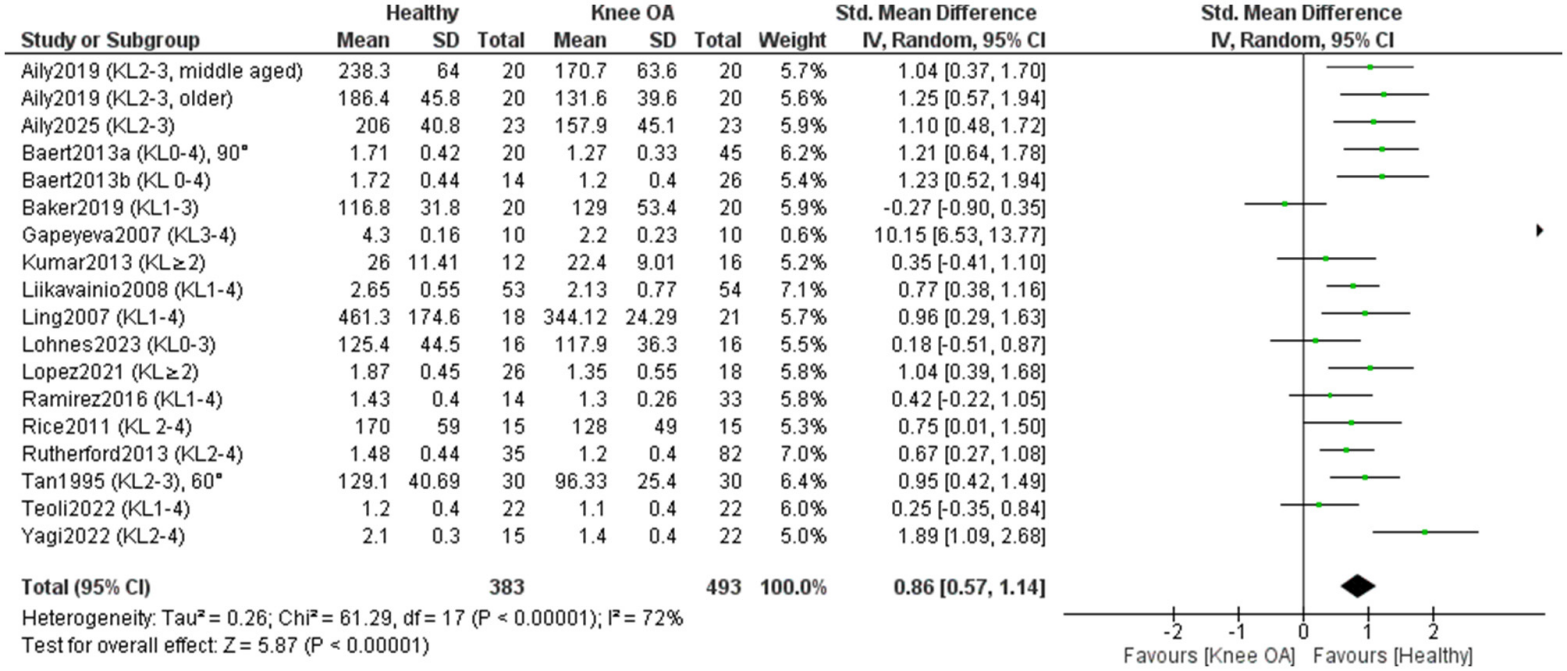
Meta-analysis of differences in isometric knee extensor strength between individuals with knee osteoarthritis and healthy controls.

Seventeen studies included in the meta-analysis (493 KOA; 383 controls) (11, 16–18, 34, 38–42, 45–48, 51–53) examined differences in isometric knee extensor strength between individuals with KOA and healthy controls (Figure 5). Overall, the results showed a significant (*P* < 0.00001) and large effect in favor of healthy individuals [SMD = 0.86, 95% CI (0.57, 1.14)]. One study (48) reported an especially large positive effect. When this study was excluded, the effect size remained large [SMD = 0.80, 95%CI (0.58, 1.02)] and statistically significant (*P* < 0.00001), while heterogeneity decreased from I^2^ = 72% to I^2^ = 55%. A sensitivity analysis was conducted by excluding studies that did not clearly report whether measurements in KOA participants were taken from the affected limb or failed to explicitly confirm that control participants were free of KOA (39, 51, 53). In this analysis, heterogeneity increased to I^2^ = 76%, and the effect size remained large [SMD = 0.84, 95% CI (0.49, 1.18)] and statistically significant (*P* < 0.00001). Two studies (33, 36) could not be included in the meta-analysis due to the absence of precise numerical data. However, the authors consistently reported reduced isometric knee extensor strength in individuals with KOA compared to healthy controls (33, 36). The certainty of evidence was low. Fifteen studies were rated as high quality and one as moderate quality according to the JBI checklist. Visual inspection of the funnel plot suggests no substantial asymmetry, indicating a low risk of publication bias. Most studies were symmetrically distributed around the pooled effect size, with no evidence of systematically missing small studies.

**Figure 6:**
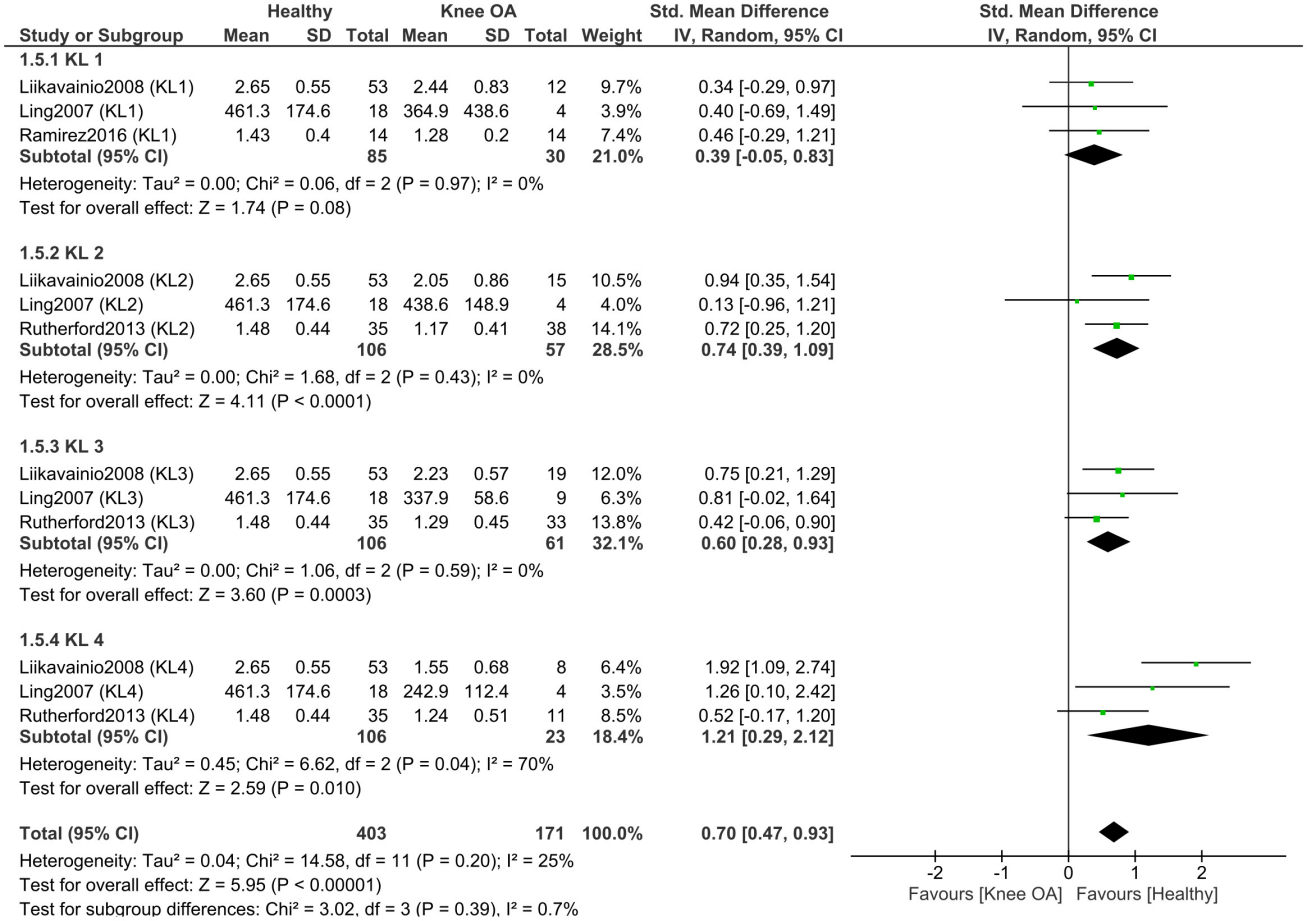
Meta-analysis of isometric knee extensor strength differences according to Kellgren–Lawrence grades of osteoarthritis severity. The total number of controls displayed in the forest plot reflects repeated use of the same control samples across Kellgren–Lawrence grade subgroups; the number of unique healthy control participants included in this analysis was 120.

To explore the impact of osteoarthritis severity, we conducted a separate meta-analysis comparing isometric knee extensor strength between healthy controls and individuals with KOA, stratified by KL grade (Figure 6). This meta-analysis included four studies (171 KOA; 120 controls) (11, 47, 51, 52). The results showed that subgroup differences were not statistically significant (*P* = 0.39), indicating that individuals with KOA, regardless of KL grade, exhibit a similar reduction in isometric knee extensor strength compared to healthy controls. When examining the overall effect within each subgroup, significant differences in isometric knee extensor strength between healthy individuals and those with KOA were observed in participants with KL grades 2–4 (*P* < 0.05), but not in those with KL grade 1 (*P* = 0.08). The effect size increased with higher KL grades, with the largest effect observed in the KL 4 subgroup (SMD = 1.21).

**Figure 7:**
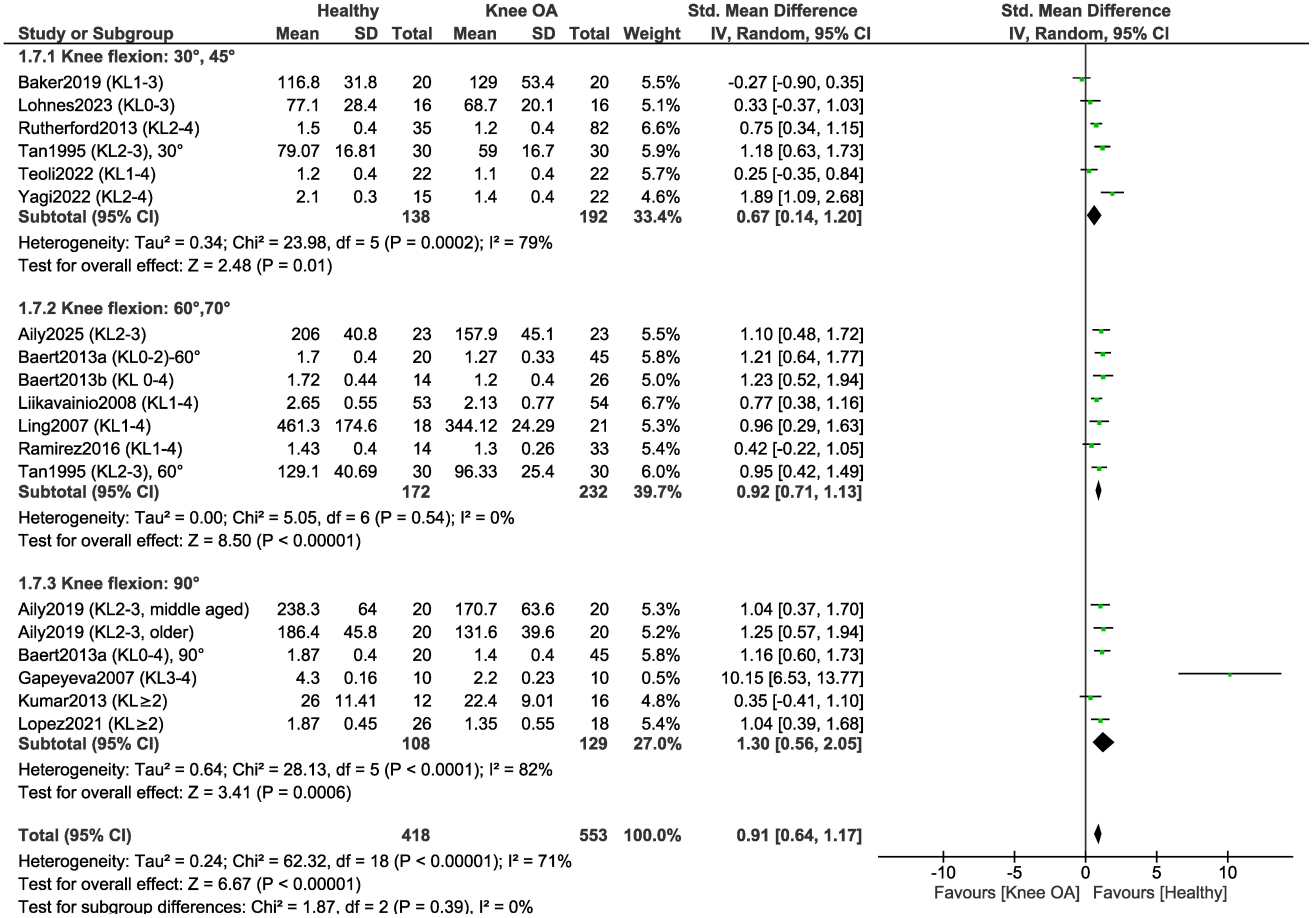
Meta-analysis of isometric knee extensor strength differences according to the knee flexion angle during strength assessment.

To determine whether isometric knee extensor strength differs between individuals with KOA and healthy controls depending on the knee flexion angle during testing, we conducted a separate meta-analysis (Figure 7). The analysis revealed no statistically significant subgroup differences based on the knee flexion angle (*P* = 0.39). These findings indicate that the magnitude of the strength deficit in individuals with KOA, compared to healthy controls, does not substantially vary across different knee flexion angles during isometric testing. The effect size increased with greater knee flexion angles, with the largest effect observed at 90 ° of knee flexion (SMD = 1.30), indicating that the strength differences between healthy individuals and those with KOA are more pronounced when measurements are taken in a more flexed knee position. Even after removing the study that showed a substantially higher positive effect and was considered an outlier (48), the subgroup with measurements taken at 90° of knee flexion still demonstrated the largest effect size (SMD = 1.01).

### 3.6 Differences in isometric knee flexor strength between individuals with knee osteoarthritis and healthy controls

**Figure 8:**
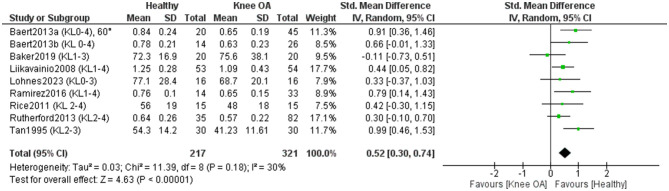
Meta-analysis of differences in isometric knee flexor strength between individuals with knee osteoarthritis and healthy controls.

Nine studies included in the meta-analysis (321 KOA; 217 controls) (11, 16, 34, 38, 41, 46, 47, 52, 53) examined differences in isometric knee flexor strength between individuals with KOA and healthy controls (Figure 8). Overall, the results showed a statistically significant (*P* < 0.00001) and moderate effect in favor of healthy individuals [SMD = 0.52, 95% CI (0.30, 0.74)]. A sensitivity analysis was performed by excluding studies that did not clearly report whether measurements in KOA participants were taken from the affected limb or did not explicitly confirm that control participants were free of KOA (52, 53). In this analysis, the effect size remained moderate [SMD = 0.42, 95% CI (0.20, 0.63)] and statistically significant (*P* < 0.0001). One study (33) also assessed isometric knee flexor strength but was not included in the meta-analysis, as the exact measurement values could not be extracted from the study. The authors reported no statistically significant differences in knee flexor strength between patients with KOA and healthy controls (*P* > 0.05) (33). Certainty of evidence was low, with most studies rated high quality and one moderate (JBI).

**Figure 9:**
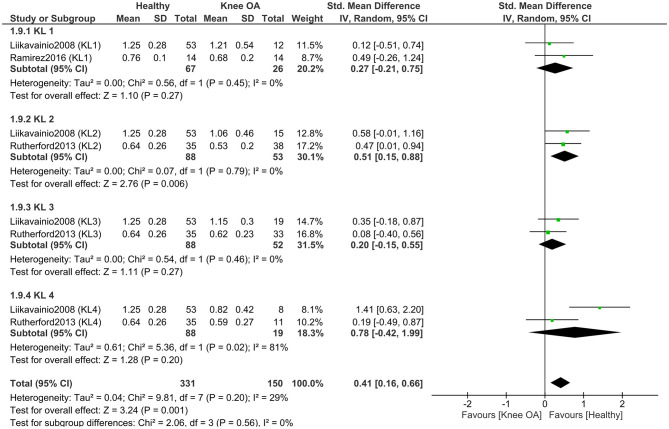
Meta-analysis of isometric knee flexor strength differences according to Kellgren–Lawrence grades of osteoarthritis severity. The total number of controls displayed in the forest plot reflects repeated use of the same control samples across Kellgren–Lawrence grade subgroups; the number of unique healthy control participants included in this analysis was 102.

A subgroup meta-analysis (3 studies; 150 KOA, 102 controls) (11, 47, 52) found no significant differences by KL grade (*P* = 0.56), indicating similar isometric knee flexor strength deficits across KOA severities (Figure 9).

### 3.7 Differences in concentric isokinetic knee extensor strength between individuals with knee osteoarthritis and healthy controls

**Figure 10:**
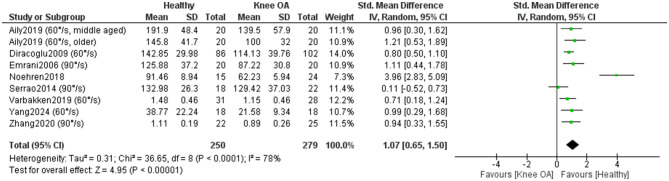
Meta-analysis of concentric isokinetic knee extensor strength differences between individuals with knee osteoarthritis and healthy controls.

Eight studies included in the meta-analysis (279 KOA; 250 controls) (32, 35, 37, 40, 50, 54–56) examined differences in concentric isokinetic knee extensor strength between individuals with KOA and healthy controls (Figure 10). Overall, the results showed a statistically significant (*P* < 0.00001) and large effect in favor of healthy individuals [SMD = 1.07, 95% CI (0.65, 1.50)]. However, heterogeneity was high (I^2^ = 78%). One study (54) stood out due to a markedly positive effect in favor of healthy individuals, while another study (55) showed a positive effect in favor of individuals with KOA. When these two studies were excluded and the meta-analysis was repeated, the overall effect remained large (SMD = 0.89) and statistically significant (*P* < 0.00001), while the heterogenity decreased significantly (I^2^=0%). A sensitivity analysis was conducted by excluding studies that did not clearly report whether measurements in KOA participants were taken from the affected limb or did not explicitly confirm that control participants were free of KOA (32, 54, 56). In this analysis, heterogeneity was moderate (I^2^ = 32%), while the effect size remained large [SMD = 0.80, 95% CI (0.53, 1.06)] and statistically significant (*P* < 0.00001). The certainty of evidence was low. All studies were rated as high quality and one as moderate quality according to the JBI checklist.

One study (36) could not be included in the meta-analysis due to the lack of precise numerical data. Nevertheless, the study also reported reduced concentric knee extensor strength in individuals with KOA, who produced 56% lower torque compared to healthy controls.

**Figure 11:**
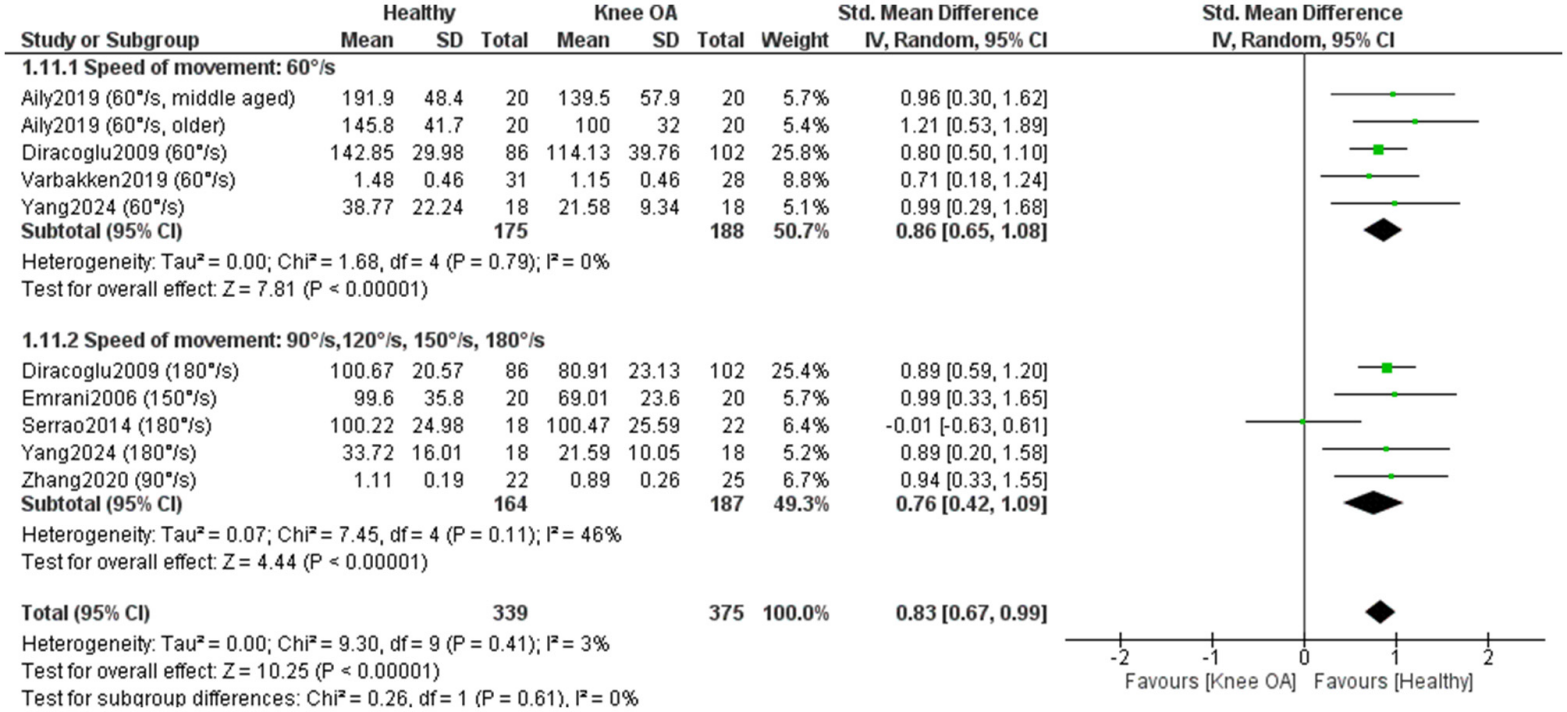
Meta-analysis of concentric isokinetic knee extensor strength differences according to speed of movement during strength assessment. The total number of controls displayed in the forest plot reflects repeated use of the same control samples across movement-velocity subgroups; the number of unique healthy control participants included in this analysis was 235.

A subgroup meta-analysis (7 studies; 375 KOA, 235 controls) (32, 35, 37, 40, 50, 55, 56) found no significant differences by angular velocity (*P* = 0.61), indicating that concentric knee extensor deficits in KOA are consistent across low and moderate testing speeds (Figure 11).

### 3.8 Differences in concentric isokinetic knee flexor strength between individuals with knee osteoarthritis and healthy controls

**Figure 12:**
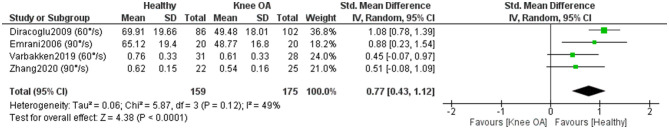
Meta-analysis of concentric isokinetic knee flexor strength differences between individuals with knee osteoarthritis and healthy controls.

Four studies included in the meta-analysis (175 KOA; 159 controls) (35, 37, 50, 56) examined differences in concentric isokinetic knee flexor strength between individuals with KOA and healthy controls (Figure 12). Overall, the results showed a statistically significant (*p* < 0.0001) and large effect in favor of healthy individuals [SMD = 0.77, 95% CI (0.43, 1.12)] with a small heterogeneity across studies (I^2^ = 49%). A sensitivity analysis was conducted by excluding studies that did not clearly report whether measurements in KOA participants were taken from the affected limb or did not explicitly confirm that control participants were free of KOA (56). In this analysis, heterogeneity was moderate (I^2^ = 53%), while the effect size remained large [SMD = 0.84, 95% CI (0.44, 1.24)] and statistically significant (*P* < 0.0001). The certainty of evidence was low. All studies were rated as high quality and one as moderate quality according to the JBI checklist.

**Figure 13:**
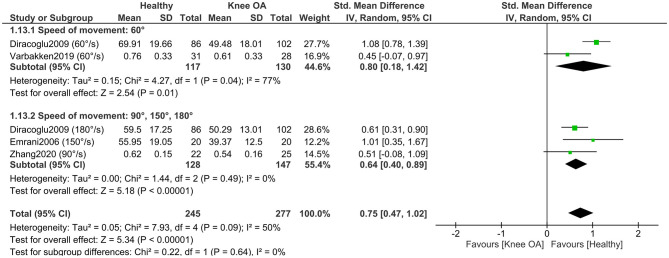
Meta-analysis of concentric isokinetic knee flexor strength differences according to speed of movement during strength assessment.

A subgroup meta-analysis (4 studies; 175 KOA, 159 controls) (35, 37, 50, 56) found no significant differences by angular velocity (*P* = 0.64), indicating similar concentric knee flexor strength deficits in KOA across low and moderate movement velocities (Figure 13).

### 3.9 Differences in eccentric isokinetic knee extensor strength between individuals with knee osteoarthritis and healthy controls

Two studies reported lower eccentric extensor strength in KOA than in healthy controls (36, 55). Hortobágy et al. (36), reported that OA patients produced 76% lower eccentric forces compared to healthy subjects. Serrão et al. (55) demonstrated significantly reduced eccentric isokinetic knee extensor strength at both 90 °/s (*P* = 0.01) and 180 °/s (*P* = 0.04) in individuals with early-stage KOA compared with healthy controls.

## 4 Discussion

This systematic review examined differences in knee joint RoM and muscle strength between individuals with and without KOA. Thirty studies were included, 28 in the meta-analysis. Results showed reduced knee flexion and extension RoM in KOA, with flexion deficits increasing with higher KL grades. Healthy controls demonstrated greater isometric, concentric, and eccentric strength of the knee extensors and flexors. Isometric strength deficits in KOA were consistent across KL grades and knee flexion angles, while concentric strength differences were similar across testing velocities.

### 4.1 Differences in knee range of motion between individuals with knee osteoarthritis and healthy controls

Deficits in knee RoM in individuals with KOA compared to healthy individuals may be attributed to a variety of factors. On one hand, KOA patients may be limited by pain or fear of pain, which prevents them from achieving a greater RoM, even if the joint itself would otherwise be capable of moving through a larger range (57, 58). On the other hand, knee RoM may be mechanically restricted by structural changes in bone and cartilage, such as the presence of osteophytes and reduced joint space (59). It can also be limited by soft tissue restrictions, including shortening or tightness of the knee flexor and extensor muscles (60, 61), knee effusion (62–64) or by excess fat, which mechanically restricts inter-segmental movement in the body (65). The latter may often contribute to limited knee RoM, as the prevalence of KOA is reported to be 3.59 times higher among individuals with obesity compared to non-obese individuals (66). It is important to recognize that all these factors restricting RoM contribute to a vicious cycle—disuse and unloading of the knee joint throughout its full RoM can, in itself, lead to articular and periarticular tissue changes, which further limit knee RoM. This is supported by studies conducted on rats, which showed that prolonged immobilization in full knee flexion led to replacement of the articular cartilage with bone tissue on the anterior region of the tibial articular surface (67, 68). It is possible that similar changes may also occur in individuals with KOA who do not load the knee in a neutral position—i.e., 0 ° of extension. This lack of full extension appears to be quite common, as all studies included in the review report mean knee extension values in individuals with KOA that do not reach 0 °.

Our meta-analysis showed that the deficit in knee RoM increases with higher KL grades. Specifically, individuals with KL grade four exhibited a flexion deficit of 27.02 degrees compared to healthy controls, while those with KL grade one had a deficit of only 2.42 degrees. Similar findings were reported by Hilfiker et al. (60), who found that radiographic severity is independently associated with deficits in both knee extension and flexion in patients with KOA. These results support the traditional biomedical model, indicating that the extent of structural pathology (degeneration) affects joint function. Additionally, this highlights the importance of early intervention: in individuals with lower KL grades, strategies should focus on preserving mobility and preventing functional decline, potentially reducing the severity of deficits as the disease progresses.

### 4.2 Differences in knee muscle strength between individuals with knee osteoarthritis and healthy controls

Our results showed that individuals with KOA have reduced isometric, concentric, and eccentric strength of the knee muscles compared to healthy individuals. Similar to deficits in knee RoM, muscle weakness can also arise from a variety of factors. Studies have shown that individuals with KOA spend approximately two-thirds of their daily time in sedentary behaviour (61). While most of the general population does not meet physical activity guidelines, people with hip or knee osteoarthritis are still approximately 25% less active than those without OA (69). Sedentary behaviour can lead to a loss of muscle mass and a reduction in muscle cross-sectional area (70) which in turn decreases the muscle's ability to generate force (71, 72). According to strength inhibition theory, peak muscle force can be inhibited by pain (73). The presence of muscle pain may reduce muscle strength due to altered reflex pathways, where nociceptive input from group III and IV afferents facilitates inhibitory effects on agonist moto neurons, thereby decreasing maximal voluntary force production (74). Altered reflex pathways can be influenced not only by pain but also by joint swelling, inflammation, joint laxity, and damage to sensory receptors (75). There is evidence that up to 50% of individuals with KOA are unable to fully activate their quadriceps muscle (76). Therefore, to improve peak muscle force in KOA patients, it is important not only to engage in strength training, but also to include interventions aimed at enhancing muscle activation, such as cryotherapy, transcutaneous electrical nerve stimulation, or neuromuscular electrical stimulation (75).

Our meta-analysis found that deficits in isometric strength between individuals with and without KOA were similar across different KL grades of KOA severity. The strength deficits did not appear to increase with higher KL grades. These findings suggest that reduced muscle strength may not be associated with structural joint changes. It is possible that the cause of reduced muscle strength lies elsewhere—perhaps in the presence of symptoms rather than structural joint changes. Previous studies have shown that only 47% of individuals with KL grade 2–4 report experiencing pain, and up to 23.5% of those with KL grade 4 report no pain (77, 78). On the other hand, our findings may indicate that muscle strength does not decline proportionally with the progression of structural joint changes.

We also found that deficits in isometric knee extensor strength in individuals with KOA compared to healthy controls were independent of the knee flexion angle used during testing, and that deficits in concentric strength were independent of movement velocity. These findings may guide future research on muscle strength assessment. Given that similar strength deficits are observed regardless of joint position and movement speed, it would be reasonable to conduct strength assessments under conditions that minimize symptoms and maximize patient comfort. We would expect greater discomfort in the 90 ° of knee flexion position, as this angle generates higher compressive forces on the tibiofemoral joint (20). It is important to keep in mind that strength measurements may be invalid if performed in the presence of pain (73), which further highlights the importance of adapting testing conditions to the patient's symptoms.

## 5 Strengths and limitations

According to the JBI scale, most of the studies included in the meta-analysis were of high methodological quality, with the majority achieving a score of seven. However, the effect sizes were highly heterogeneous in some of the meta-analyses. In the meta-analyses comparing RoM between individuals with and without KOA, we included both active and passive RoM measurements, which may have influenced the results. Additionally, the individuals with and without KOA were not matched using the same criteria across studies. If differences in age or other demographic characteristics existed between groups, they may have affected the magnitude of the observed differences. Some conclusions in the analyses are based on results observed in individuals with KOA across different KL grades. It is possible that different findings would have emerged if the analyses had been conducted separately for each KL grade. Some studies did not report whether pain was present during measurements, which could compromise the validity of the results. Only English-language publications were included, which may have led to language bias. An important limitation of this review is that balance-related outcomes were not included. Balance impairments and falls are highly prevalent in individuals with KOA and represent a major clinical concern. As balance measures were outside the scope of the present review, the relationship between knee RoM, muscle strength, and balance function could not be addressed. Another limitation is that potential regional and cultural differences in functional demands were not considered. In some regions, particularly in parts of Asia, daily activities such as dining or sitting on the floor require deep knee flexion, making knee RoM particularly important. As most included studies originated from Western countries, the functional relevance of knee flexion deficits may differ across populations, which should be considered when interpreting the findings.

## 6 Conclusions

This review confirms that individuals with symptomatic KOA have reduced knee RoM and muscle strength across all contraction types. Flexion deficits worsen with disease severity, whereas strength deficits remain stable across KL grades, suggesting links to factors such as pain or muscle inhibition rather than structural changes. Similar deficits across knee joint angles and angular velocities during testing indicate that assessment protocols can be flexibly adapted to patient comfort in clinical practice. Conclusions were drawn within the limitations of the available observational data, and no causal inferences were made. Future studies assessing knee strength and RoM should more precisely report whether participants have symptomatic KOA, whether control subjects are truly free of KOA (either symptomatically or radiographically), whether pain was present during measurements, and which leg was tested. To enable more detailed subgroup analyses, future research should also report measurement outcomes stratified by individual KL grades.

## Data Availability

Publicly available datasets were analyzed in this study. Existing datasets from previously published studies were analyzed as part of the systematic review and meta-analysis. All data were extracted from peer-reviewed articles available in the public domain.
