# Supplementary material for: Disease severity affects knee range of motion but not strength deficits in knee osteoarthritis: a systematic review and meta-analysis
Source: Front Med (Lausanne). 2026 Feb 13;13:1737973. doi: 10.3389/fmed.2026.1737973 (PMC12946006; doi:10.3389/fmed.2026.1737973)
Supplement: Supplementary file 1 [file Table_1.docx]

**Table S1.** Assessment of study quality with Joanna Briggs Institute checklist.

| Study / Score | 1. Were the criteria for inclusion in the sample clearly defined? | 2. Were the study subjects and the setting described in detail? | 3. Was the exposure measured in a valid and reliable way? | 4. Were objective, standard criteria used for measurement of the condition? | 5. Were confounding factors identified? | 6. Were strategies to deal with confounding factors stated? | 7. Were the outcomes measured in a valid and reliable way? | 8. Was appropriate statistical analysis used? | **Total points** |
| --- | --- | --- | --- | --- | --- | --- | --- | --- | --- |
| Aily et al. (2019) | Yes | Yes | Yes | Yes | Yes | Yes | Yes | Not applicable | **7** |
| Aily et al. (2025) | Yes | Yes | Yes | Yes | Yes | Yes | Yes | Not applicable | **7** |
| Baert, Mahmoudian, et al. (2013) | Yes | Yes | Yes | Yes | Yes | Yes | Yes | Not applicable | **7** |
| Baert, Jonkers, et al. (2013) | Yes | Yes | Yes | Yes | Yes | Yes | Yes | Not applicable | **7** |
| Diracoglu et al. (2009) | Yes | Yes | Yes | Yes | Yes | Yes | Yes | Not applicable | **7** |
| Gapayeva et al. (2007) | Yes | Yes | Yes | Yes | Yes | Yes | Yes | Not applicable | **7** |
| Kocak et al. (2009) | Yes | Yes | Yes | Yes | Yes | Yes | Yes | Not applicable | **7** |
| Kumar et al. (2013) | Yes | Yes | Yes | Yes | Yes | Yes | Yes | Not applicable | **7** |
| Liikavainio et al. (2008);  Lyytinen et al. (2010) | Yes | Yes | Yes | Yes | Yes | Yes | Yes | Not applicable | **7** |
| Ling et al. (2007) | Yes | Yes | Yes | Yes | Yes | Yes | Yes | Not applicable | **7** |
| Rodriguez‐Lopez et al. (2021) | Yes | Yes | Yes | Yes | Yes | Yes | Yes | Not applicable | **7** |
| Ramirez et al. (2016) | Yes | Yes | Yes | Yes | Yes | Yes | Yes | Not applicable | **7** |
| Rutherford et al. (2013) | Yes | Yes | Yes | Yes | Yes | Yes | Unclear | Not applicable | **6** |
| Tan et al. (1995) | No | Yes | Yes | Yes | Yes | Unclear | Yes | Not applicable | **5** |
| Varbakken et al. (2019) | Yes | Yes | Yes | Yes | Yes | Yes | Yes | Not applicable | **7** |
| Yagi et al. (2022) | Yes | Yes | Yes | Yes | Yes | Yes | Yes | Not applicable | **7** |
| Yang et al. (2024) | No | Yes | Yes | Yes | Yes | Unclear | Yes | Not applicable | **5** |
| Baker et al. (2019) | Yes | Yes | Yes | Yes | Yes | Yes | Yes | Not applicable | **7** |
| Childs et al. (2004) | Yes | Yes | Yes | Yes | Yes | Yes | Unclear | Not applicable | **6** |
| Emrani et al. (2006) | Yes | No | Yes | Yes | Yes | Yes | Yes | Not applicable | **6** |
| Hortobagy et al. (2004) | Yes | Yes | Yes | Yes | Yes | Unclear | Yes | Not applicable | **6** |
| Lohnes et al. (2023) | Yes | Yes | Yes | Yes | Yes | Yes | Yes | Not applicable | **7** |
| Noehren et al. (2018) | No | Yes | Yes | Yes | Yes | Yes | Yes | Not applicable | **6** |
| Rice et al. (2011) | Yes | Yes | Yes | Yes | Yes | Yes | Unclear | Not applicable | **6** |
| Serrao et al. (2015) | Yes | Yes | Yes | Yes | Yes | Yes | Yes | Not applicable | **7** |
| Teoli et al. (2022) | Yes | Yes | Yes | Yes | Yes | Yes | Yes | Not applicable | **7** |
| Ucurum et al. (2024) | Yes | Yes | Yes | Yes | Yes | Yes | Yes | Not applicable | **7** |
| Yagi et al. (2023) | Yes | Yes | Yes | Yes | Yes | Unclear | Yes | Not applicable | **6** |
| Zhang et al. (2020) | No | Yes | Yes | Yes | Yes | Yes | Yes | Not applicable | **6** |
